# Reconstruction of chronic proximal hamstring tear with hamstring autograft—hamstring on hamstring technique: technical note

**DOI:** 10.1093/jhps/hnaf060

**Published:** 2025-09-30

**Authors:** Raquel Lima Cunha, Alexandre Castro, Manuel Padin, Francisco Alpoim, José Manuel Teixeira, Jorge Cruz de Melo, Fernando Leal

**Affiliations:** Department of Orthopaedics and Traumatology, Unidade Local de Saúde de Entre o Douro e Vouga, Rua Dr. Cândido de Pinho, 4520-211 Santa Maria da Feira, Portugal; Department of Orthopaedics and Traumatology, Unidade Local de Saúde de Entre o Douro e Vouga, Rua Dr. Cândido de Pinho, 4520-211 Santa Maria da Feira, Portugal; Department of Orthopedics and Traumatology, Hospital da Luz Arrábida, PCT de Henrique Moreira 150, 4400-346 Vila Nova de Gaia, Porto, Portugal; Department of Orthopedics and Traumatology, Hospital da Luz Arrábida, PCT de Henrique Moreira 150, 4400-346 Vila Nova de Gaia, Porto, Portugal; Fidelidade Insurance Company – Clinical Department of Work Related Accidents Assistance, R. Direita das Campinas 324, 4100-207, Porto, Portugal; Department of Orthopedics and Traumatology, Hospital da Luz Arrábida, PCT de Henrique Moreira 150, 4400-346 Vila Nova de Gaia, Porto, Portugal; Fidelidade Insurance Company – Clinical Department of Work Related Accidents Assistance, R. Direita das Campinas 324, 4100-207, Porto, Portugal; Department of Orthopedics and Traumatology, Hospital da Luz Arrábida, PCT de Henrique Moreira 150, 4400-346 Vila Nova de Gaia, Porto, Portugal; Fidelidade Insurance Company – Clinical Department of Work Related Accidents Assistance, R. Direita das Campinas 324, 4100-207, Porto, Portugal; Department of Orthopedics and Traumatology, Hospital da Luz Arrábida, PCT de Henrique Moreira 150, 4400-346 Vila Nova de Gaia, Porto, Portugal; Fidelidade Insurance Company – Clinical Department of Work Related Accidents Assistance, R. Direita das Campinas 324, 4100-207, Porto, Portugal

## Abstract

Chronic proximal hamstring tears, often resulting from sports-related trauma or repetitive strain, present a challenging clinical scenario characterized by long-standing tendon damage and functional impairment. These injuries, particularly common in the fourth and fifth decades of life, significantly affect an individual’s quality of life and athletic performance and require effective treatment strategies. Surgical intervention is indicated when conservative treatments fail, with the primary goal of restoring the mechanical function of the hamstrings. Traditional primary repair may not be feasible in chronic cases with tendon retraction and muscle atrophy. Surgical reconstruction techniques aim to restore tendon integrity and improve symptoms. This technical note describes a surgical technique using gracilis and semitendinosus autografts from the contralateral leg to repair chronic proximal hamstring tears. This approach aims to reinforce the reconstruction and overcome the limitations of direct repair in chronic cases, with the aim of improving strength and durability, offering a promising alternative for patients with significant tendon retraction. Reconstruction of chronic proximal hamstring tears with autografts represents an innovative approach to a challenging clinical problem. This technique offers potential benefits in strength and function restoration, with careful patient selection and surgical technique being crucial for successful outcomes. As evidence for this approach evolves, it may become a valuable tool in managing chronic proximal hamstring tears.

## INTRODUCTION

Chronic proximal hamstring tears are a difficult clinical issue marked by long-term tendon damage and functional impairment, often due to sports-related trauma or repetitive strain [1, 2]. These injuries can significantly impact an individual’s quality of life and athletic performance [1, 2]. Complete tears are uncommon, usually occurring at the myotendinous junction, with about 12% of avulsion injuries originating from the ischial tuberosity [3]. In non-sports populations, especially in the fourth or fifth decades of life, these injuries often result from eccentric trauma, leading to tendon avulsion with significant tendon retraction (complete rupture with retraction higher than 5 cm—Woods 5) [1, 4].

Traditional surgical techniques have focused on restoring tendon integrity and relieving symptoms [1, 4]. Early surgical repair is typically recommended, but if treatment is delayed, the injury may become chronic, leading to tendon retraction and muscle atrophy, making direct repair challenging [1, 4]. Patients with chronic injuries often suffer from ongoing weakness, require walking aids, and struggle to return to pre-injury activity levels [2, 5]. In patients with chronic tears classified as Woods 5 (complete avulsion with significant retraction)—particularly those experiencing persistent pain for more than 3 months, neglected tears or failed previous repairs—at this point, primary repair is not possible, and reconstruction should be strongly considered [2, 4, 5]. The indication for reconstruction is especially recommended in cases with tendon gaps exceeding 6 cm, accompanied by muscle belly atrophy. A more complex approach has emerged for reconstruction of chronic proximal hamstring tears with graft [2, 5]. The majority of described techniques utilize an Achilles tendon allograft. However, alternative graft options, including ipsilateral distal hamstring autografts and ipsilateral fascia lata autografts, have also been reported [1, 4].

In this technical note, we described a surgical technique using gracilis and semitendinous tendons autograft from contralateral leg to repair chronic proximal hamstring tear. This technique enable the repair of the damaged tendons when the lesion gap impairs direct repair and allows also reinforcing the repair. While the evidence base for this specific approach is evolving and may not be as extensive as that for traditional primary reconstruction, it holds promise in enhancing the strength and durability of the repair [2, 5].

This work outlines the current knowledge on reconstructing chronic proximal hamstring tears with grafts and details of our proposed technique for reconstruction.

## CLINICAL PRESENTATION AND DIAGNOSIS

Patients with chronic proximal hamstring tears typically present with persistent pain, weakness, and functional limitations in the hip and thigh, often after activities like sprinting or jumping [2, 6, 7]. Normally these patients present as an initial neglected lesion or after conservative treatment of previously documented lesion. In most cases, symptoms persist for >12 weeks, indicating a chronic condition [2, 7, 8]. Pain and discomfort are often felt in the posterior thigh and may radiate down the leg, impairing the gait [2, 7, 8]. Patients may also report difficulty sitting, walking, or engaging in activities involving hip extension, as well as symptoms of sciatic nerve irritation [2, 7, 8].

A thorough physical examination is crucial for assessing the extent of the tear and guiding treatment [7]. This includes checking for an antalgic gait, tenderness, a palpable defect near the ischial tuberosity, and evaluating hip and knee range of motion [7]. Imaging studies, particularly magnetic resonance image, are often used to confirm the diagnosis, assess tendon retraction, and plan for potential surgery [7].

## SURGICAL TECHNIQUE

### Patient positioning

The patient is first positioned supine to harvest the hamstrings from the contralateral leg, then placed in a prone position to expose the posterior gluteal area.

### Surgical approach and reconstruction

A vertical incision is made from the ischial tuberosity to the retracted musculotendinous complex (Fig. 1)—this approach provides adequate exposure for releasing scar tissue and avoiding injury to the sciatic nerve. Then subcutaneous dissection should be performed carefully to identify and avoid lesion of the posterior femurocutaneous nerve. Additionally, the sciatic nerve should be identified and released from surrounding scar tissue (Fig. 2). After releasing adhesions, the remaining gap between the ischial tuberosity and the mobilized stump hamstring is measured with the knee positioned at 30°–40° of flexion (Fig. 2).

**Figure 1:**
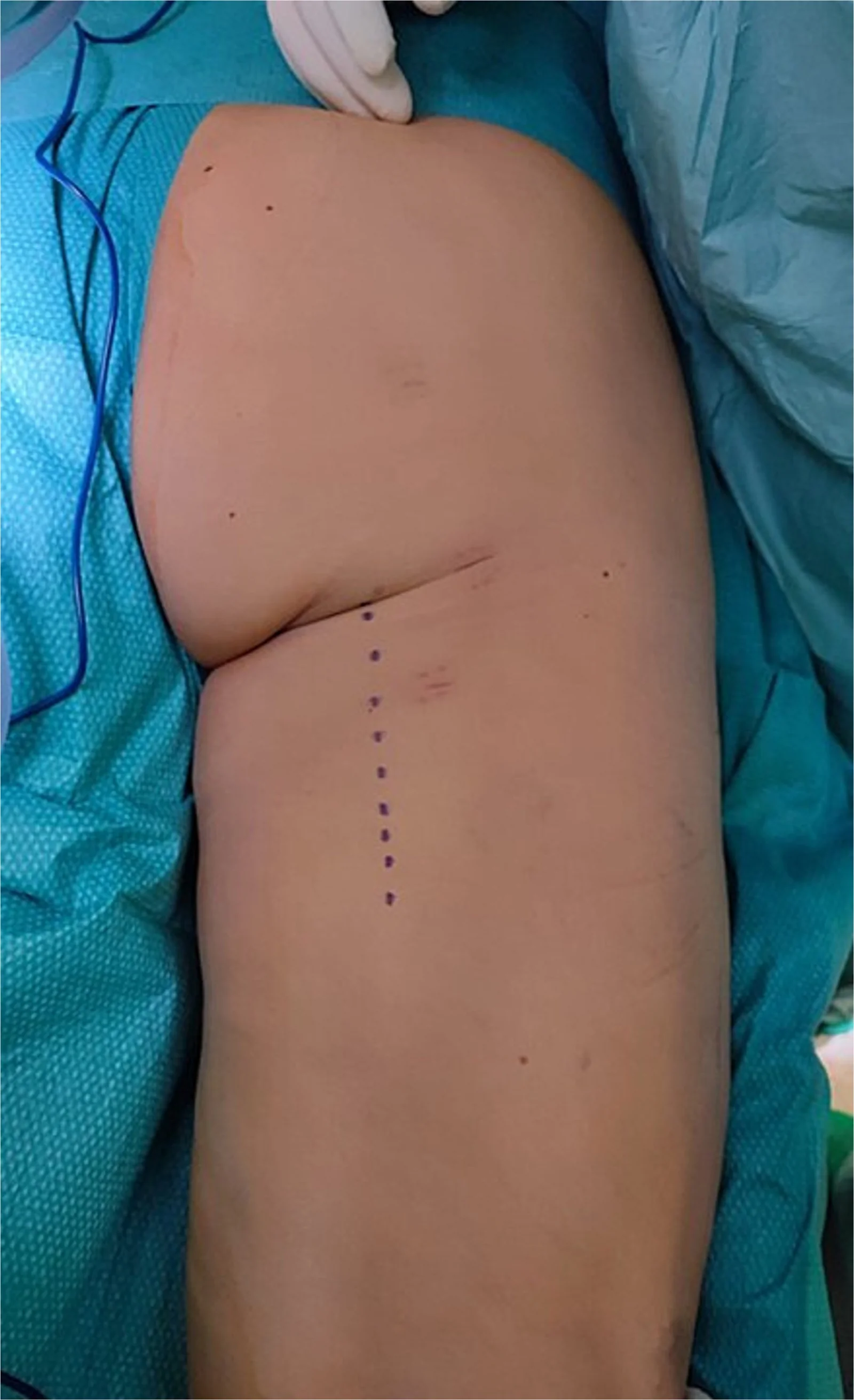
The patient placed in the prone position, exposing the posterior gluteal fold. A vertical longitudinal incision from the ischial tuberosity (palpated immediately proximal to the gluteal fold) to the retracted musculotendinous complex.

**Figure 2:**
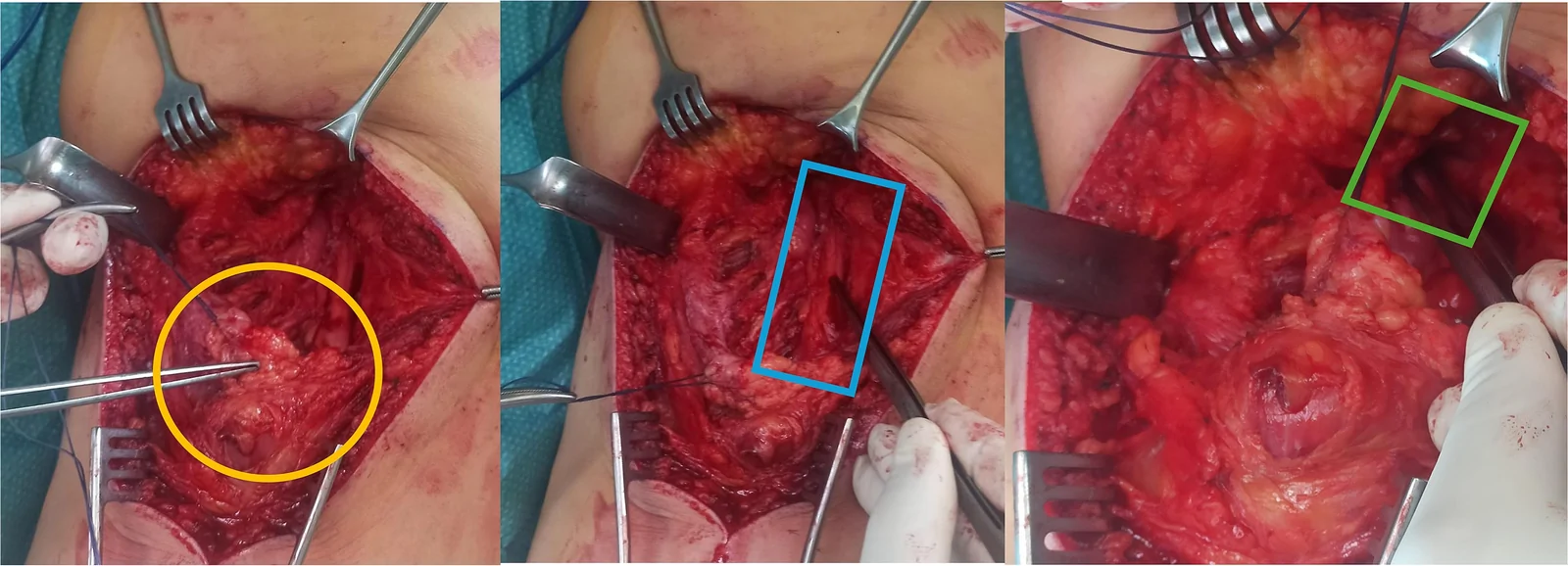
Intraoperative image demonstrating the release of adhesions surrounding the retracted tendon stump from proximal hamstring facilitating maximal tendon excursion (yellow circle). The sciatic nerve must be identified and released laterally from the hamstring stump and associated fibrous scar tissue (blue rectangle). The ischial tuberosity should then be exposed and prepared, with cruentation and removal of all soft tissue footprint in the ischial tuberosity (green square).

The gracilis and semitendinosus tendons, harvested from the contralateral leg, should be prepared (gracilis and semitendinosus are kept attached together and both ends are sutured with high-resistance sutures) to form a single tendon (Fig. 3). The graft is passed through the tendon stump in a Pulvertaft weave fashion (are folded in half and sutured the two stumps together with krackow sutures) and anchored to this native hamstring bundle with interrupted sutures (Figs 4 and 5). The ischial tuberosity should then be exposed and prepared, with cruentation and removal of all soft tissue footprint in the ischial tuberosity. Two to three anchors are placed at the ischium footprint and the sutures from each anchor are threaded through the graft. At this point, the knee is kept in 90° of flexion for adequate tensioning of the graft hamstring tendon, with each suture being tensioned, reducing the autograft to the ischium. Then moved through a 30°–40° of flexion to tie every suture anchor. The knee is taken through a 30° arc of motion while assessing for appropriate excursion of the hamstrings with no overdue tension or gapping at the repair site, additional passes are made through the stump, and the remaining sutures are finalized (Fig. 6). Subsequently, a suture of skin is made by planes.

**Figure 3:**
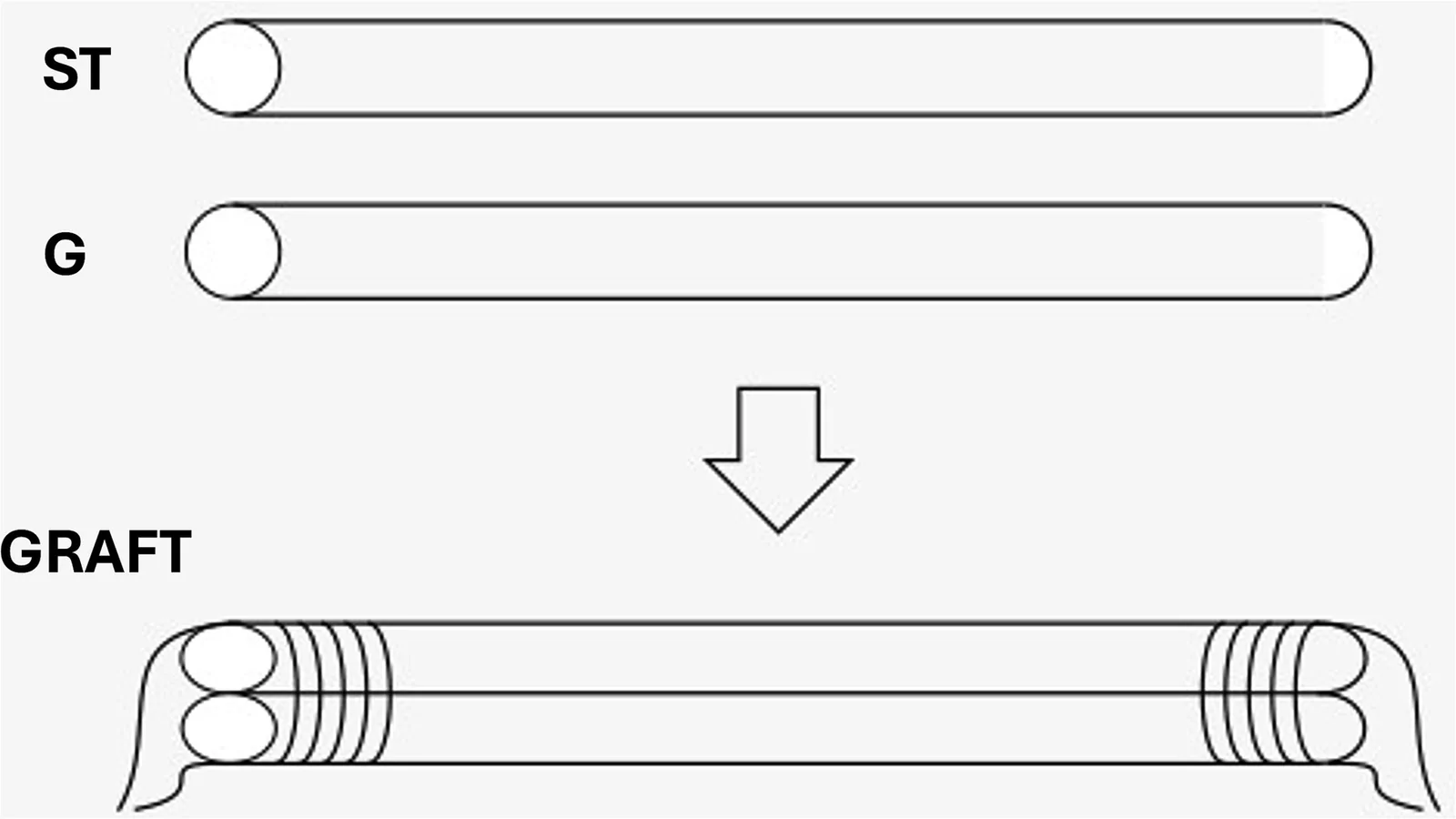
Graft preparation—gracilis and semitendinosus are kept attached together and both ends are sutured with high-resistance sutures—to form a single tendon.

**Figure 4:**
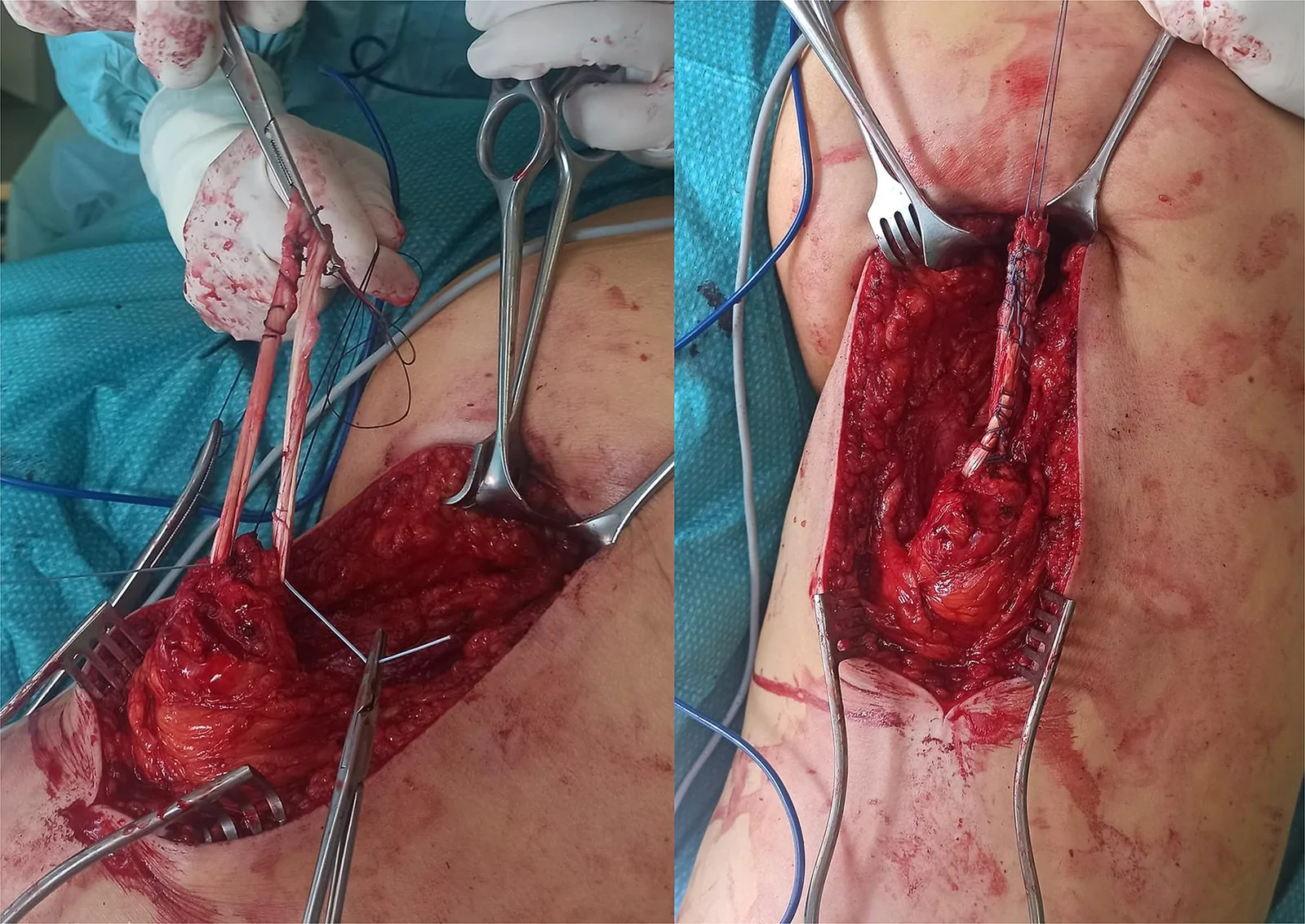
Intraoperative image showing hamstrings tendons autograft is passed through the tendon stump in a Pulvertaft weave fashion—are folded in half and sutured the two stumps together with krackow sutures—and anchored to this native hamstring stump with interrupted sutures.

**Figure 5:**
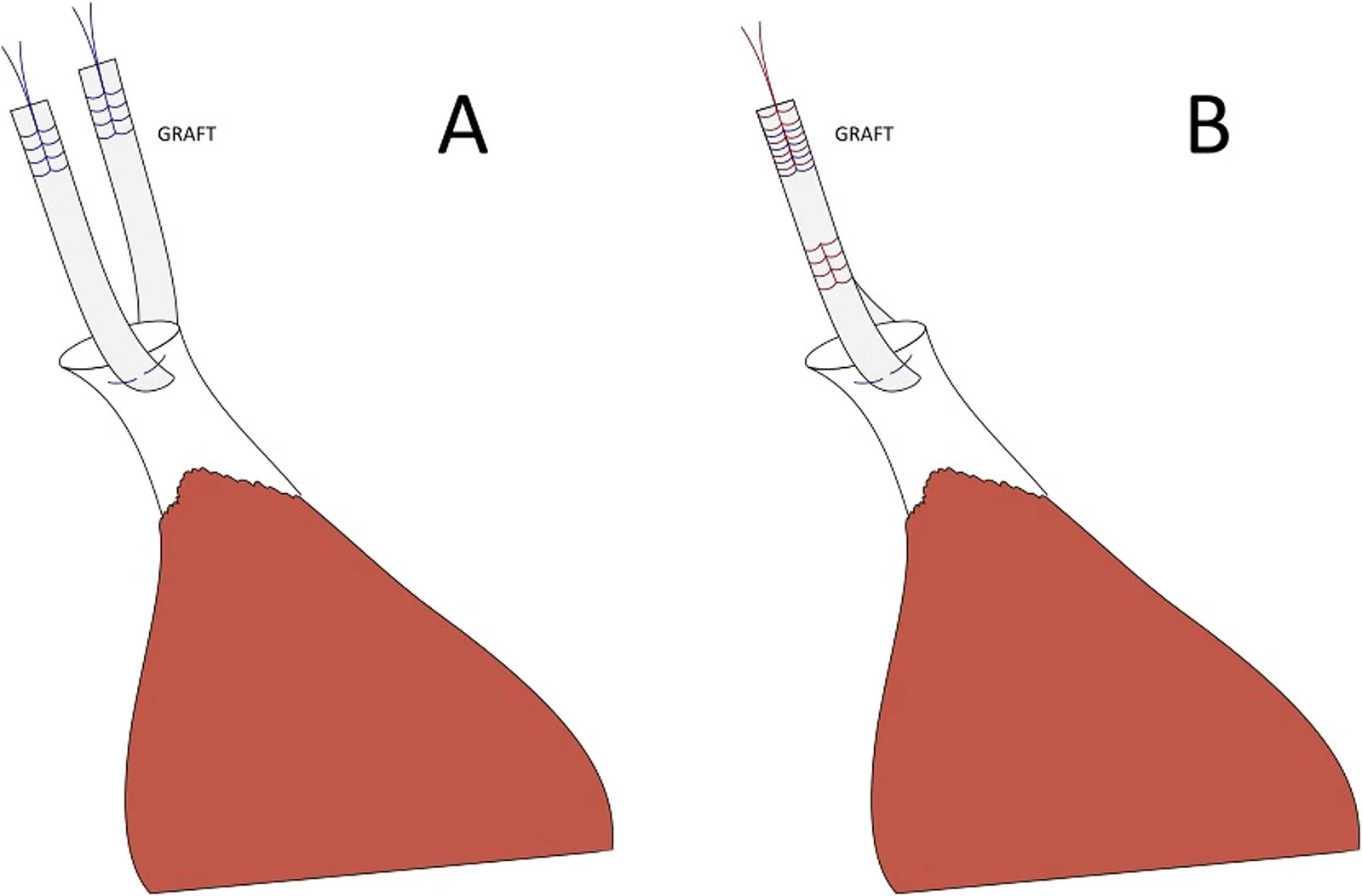
(A) Schematic representation how autograft is passed through the tendon stump in a Pulvertaft weave fashion and are folded in half. (B) Schematic representation how the two stumps are sutured together with krackow sutures and anchored to the native hamstring stump with interrupted sutures.

**Figure 6:**
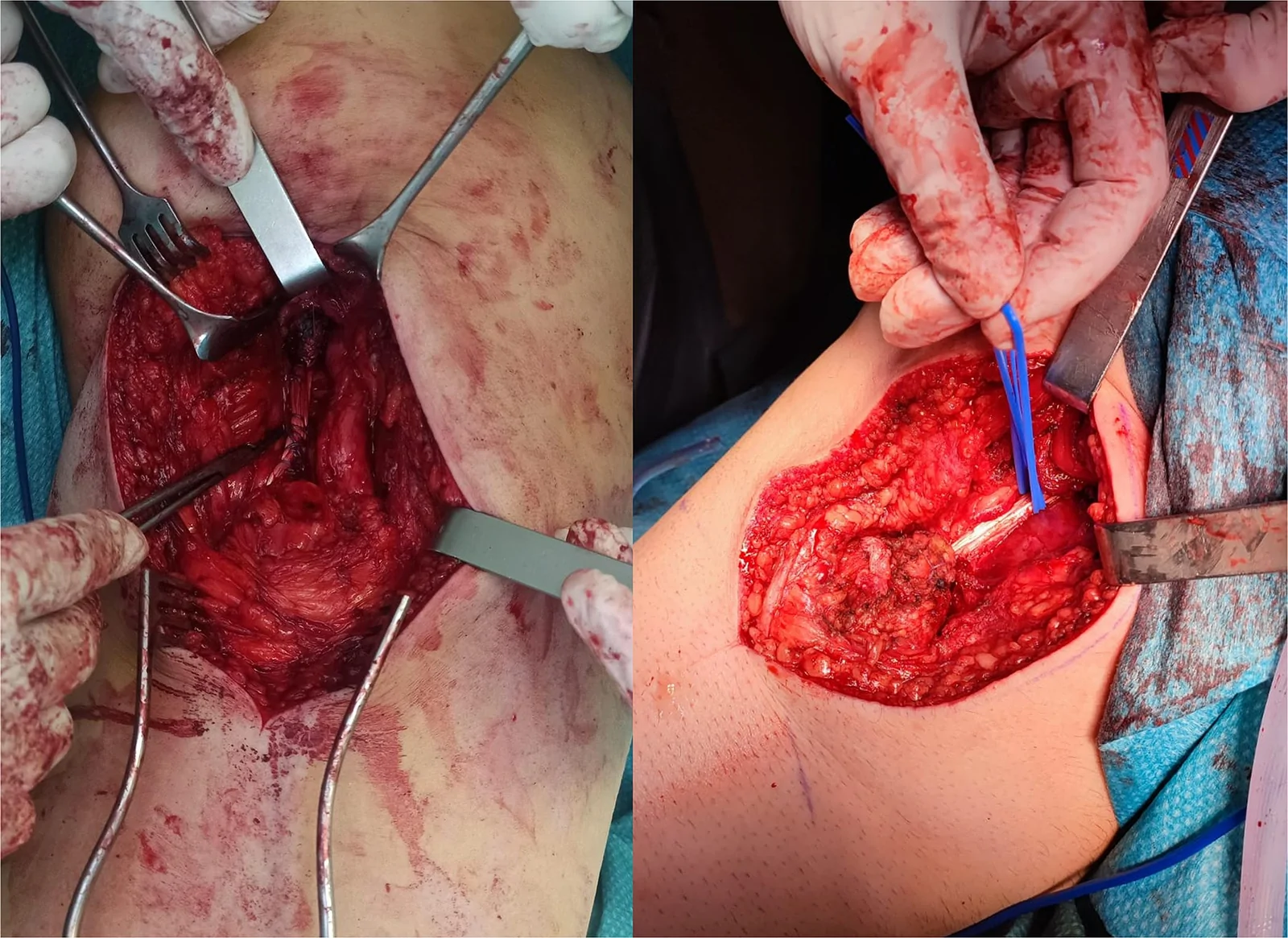
Intraoperative image showing two examples of final aspect of reconstruction of chronic proximal hamstring tear with hamstring autograft.

## Postoperative rehabilitation protocol

Postoperatively, the patient is immobilized in a brace with the knee locked at 45° of flexion for 4 weeks. Active hip flexion with knee extension is avoided to prevent stress on the reconstruction. Weight-bearing is restricted to toe-touch for the initial four weeks, followed by a gradual progression to full weight-bearing with crutches. Physiotherapy is initiated at 8 weeks post-surgery.

## DISCUSSION

Chronic proximal hamstring tears present a complex clinical challenge, often resulting from sports-related trauma or fall with eccentric mechanisms, that can severely impact an individual’s quality of life and functional capacity [4, 7]. Surgical intervention is generally recommended when all three tendons are involved, when there is significant retraction, or when conservative treatment fails [3]. Acute repairs within 6 weeks typically yield better outcomes, nevertheless patient satisfaction is still comparable with delayed repair and reconstruction for chronic injuries with tendon retraction, although require more complex surgical approaches [2, 9]. The most common surgical techniques have been a mainstay in addressing these injuries, mostly through primary repair. That can be achieved with open surgery and more recently with endoscopic surgery [2, 6]. However, when tears evolve into chronic injuries with tendon retraction, and with a large gap and muscle atrophy direct repair it is not possible, and innovative approaches are required to restore strength and function. The final goal is to restore the mechanical capability of the hamstrings to generate strength again. Though more challenging from a technical standpoint, surgical intervention for chronic tears remains a viable option with favorable outcomes. Most of techniques described involve the use of Achilles tendon allograft, but other grafts have been described such as ipsilateral distal hamstring autograft or ipsilateral fascia lata autograft. Reconstruction using a graft, particularly with an autograft from the gracilis and semitendinosus tendons, has emerged as a promising solution for chronic injuries [1, 2].

Techniques like the one described, which involve the use of autografts from the contralateral side eliminates the dependence on allografts, thereby avoiding risks related to immunological rejection, disease transmission, and limited graft availability. Furthermore, autografts demonstrate superior biological integration, lower complication rates, and greater predictability of clinical outcomes, as the graft originates from the patient’s own tissue [10–12]. The use of hamstring tendons, particularly the semitendinosus and gracilis, as autografts is widely regarded as a safe and reliable option, with relatively low donor site morbidity and minimal long-term functional deficits with most patients experiencing only mild, short-term discomfort or temporary weakness in knee flexion, which typically resolves [11, 12].

This method not only allows for the repair of the damaged tendons but also reinforces the repair, potentially reducing the risk of re-rupture and improving long-term outcomes [1, 2, 9]. While evidence for this technique is still evolving, it offers significant advantages in restoring the mechanical capability of the hamstrings and improving function in patients who cannot undergo primary repair [7, 9].

## CONCLUSIONS

The reconstruction of chronic proximal hamstring tears with autografts represents an innovative approach to a challenging clinical problem. This technique offers potential benefits in terms of integration, strength, durability, and functional recovery. Proper patient selection, thorough diagnostic evaluation, and meticulous surgical technique are critical for achieving successful outcomes. As more evidence becomes available, this approach may become an increasingly important tool in managing chronic proximal hamstring tears.

Conflict of interest: The authors declare that they have no conflicts of interest relevant to the content of this review.

## FUNDING

None declared.
